# Antioxidant and Metal Chelation-Based Therapies in the Treatment of Prion Disease

**DOI:** 10.3390/antiox3020288

**Published:** 2014-04-21

**Authors:** Marcus W. Brazier, Anthony G. Wedd, Steven J. Collins

**Affiliations:** 1 Department of Pathology, University of Melbourne, Parkville, VIC 3010, Australia; E-Mail: brazier@unimelb.edu.au; 2 The Bio21 Molecular Science and Biotechnology Institute, The University of Melbourne, Victoria 3010, Australia; E-Mail: agw@unimelb.edu.au; 3 School of Chemistry, The University of Melbourne, Victoria 3010, Australia

**Keywords:** amyloid, antioxidant, CJD, chelation, Cu, hydroxyl radical, Mn, oxidative stress, SOD2, superoxide dismutase, therapy, transmissible spongiform encephalopathy

## Abstract

Many neurodegenerative disorders involve the accumulation of multimeric assemblies and amyloid derived from misfolded conformers of constitutively expressed proteins. In addition, the brains of patients and experimental animals afflicted with prion disease display evidence of heightened oxidative stress and damage, as well as disturbances to transition metal homeostasis. Utilising a variety of disease model paradigms, many laboratories have demonstrated that copper can act as a cofactor in the antioxidant activity displayed by the prion protein while manganese has been implicated in the generation and stabilisation of disease-associated conformers. This and other evidence has led several groups to test dietary and chelation therapy-based regimens to manipulate brain metal concentrations in attempts to influence the progression of prion disease in experimental mice. Results have been inconsistent. This review examines published data on transition metal dyshomeostasis, free radical generation and subsequent oxidative damage in the pathogenesis of prion disease. It also comments on the efficacy of trialed therapeutics chosen to combat such deleterious changes.

## 1. Introduction

**Figure 1:**
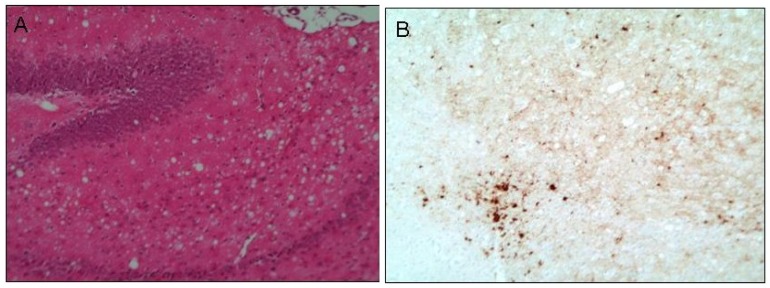
Histological examination of prion infected brain tissue. The micrograph in figure A displays the extensive vacuolation commonly referred to as spongy change, here observed at the terminal stage of prion disease. This is an example of diseased hippocampal tissue obtained from a mouse model of human prion (M1000) infection [17] stained with haematoxylin and eosin. Micrograph B shows the thalamic region, adjacent to the hippocampus, of these diseased mice depicting aggregates of prion protein in the form of plaques (dark brown deposits representing immunohistochemical detection of formic acid/4 M guanidine thiocyanite-stable PrP). Original magnification 20×.

Prion diseases, also known as transmissible spongiform encephalopathies (TSE), are rare neurodegenerative disorders delineated by the transmissible nature of the disease and the characteristic sponge-like appearance of diseased brain upon histological examination. This spongiform change is the result of extensive vacuolation predominantly in the neuropil (Figure 1A) and is reminiscent of the vacuolation observed in the brains of a mouse model of copper deficiency [1] and a manganese-superoxide dismutase (Mn-SOD2)-knockout mouse model of oxidative stress [2]. The majority of prion diseases occur sporadically through unknown mechanisms although both acquired and genetically determined forms are recognised [3]. Genetic prion disease due to single point mutations in the gene for the prion protein, *PRNP*, can lead to vastly different disease phenotypes, including the area of the brain in which infection proliferates to the greatest extent [4]. A normal polymorphism at codon 129 of the *PRNP* gene influences the phenotype resulting from a D178N mutation wherein the D178N-129M haplotype causes fatal familial insomnia, with pathology relatively restricted to the thalamus. In addition, familial Creutzfeldt-Jacob disease (CJD) with more wide-spread damage to the brain occurs in individuals carrying D178N-129V [5].

Cumulative scientific data supports that the infectious agent (“prion”) in TSE comprises aberrant misfolded conformers (termed PrP^Sc^) of the normal prion protein (PrP^C^). The PrP^C^ conversion process most likely requires additional co-factors for efficient transmission and propagation of the misfolded protein [6,7]. PrP^C^ is normally found in the outer aspect of cell membranes attached with a glycosyl-phosphatidylinositol anchor. It is widely expressed but the highest levels are found in the central nervous system which may explain why PrP^Sc^ propagation and pathogenesis is most evident in the brain.

While the prion protein and prion diseases have been studied intensively, the pathogenetic mechanisms involved in TSE are still not fully understood. For prion disease and other neurodegenerative disorders, such as Alzheimer’s, Parkinson’s and Huntington’s diseases, protein aggregation is a common pathological feature [8,9,10,11]. In addition, many studies have demonstrated that reactive oxygen/nitrogen species and heightened oxidative stress contribute to the pathogenesis of these diseases and of prion disease, in particular [12,13,14,15,16,17]. Transition metal ions can generate oxygen and nitrogen radicals via Fenton and Haber-Weiss chemistries. Such redox catalysis follows from the ability of the metals to vary their valence states (*i.e.*, gain or lose electrons) if they become “free” (*i.e.*, unconstrained by their natural ligands) [18]. The fact that elevated concentrations of transition metals have been consistently found in prion disease brain tissue suggests the potential for such catalysis (gain of function).

This review will examine the available published data on the pathological significance of changes to the normal distribution of transition metal ions. It will scrutinise the evidence for generation of toxic free radicals and reactive oxygen and nitrogen species in the brains of experimental animals and patients infected with prion disease. The efficacy of trialed therapeutic regimens designed to prevent this type of radical damage are then discussed.

## 2. Oxidative Stress

The term “oxidative stress” refers to a pathological state wherein elevated production of cellular free radicals (molecules with unpaired electrons) or other reactive species (such as hydrogen peroxide) in the cell and/or possible reduced antioxidant capacity leads to oxidative or nitrative damage to cellular constituents. Reactive oxygen or reactive nitrogen species are terms used to describe both the radical and closed shell derivatives based on those elements (Table 1). These oxidants vary in their level of reactivity: for example, the superoxide and nitric oxide radicals as well as hydrogen peroxide are relatively unreactive in aqueous solutions compared to the hydroxyl radical [18]. Oxygen and nitrogen radicals are generated during normal metabolic processes such as energy transduction through the mitochondrial electron transport chain [18]. The highly reactive nature of these species renders them potentially toxic to cells as they can readily oxidise key residues in biomolecules such as proteins, membrane lipids and nucleic acids. They can also induce breakage of amino acid and nucleic acid polymeric chains. Such chemical modification to these cellular macromolecules usually results in the loss of their normal function.

## 3. Reactive Transition Metal Ions

Transition metal atoms (the d-block elements) generally contain electrons in their outermost *d* and *s* shell orbitals. They readily lose electrons to form positively charged ions (cations) that bind to ligands to form molecules. These often feature an incomplete shell of *d* electrons (<10) with one or more unpaired electrons. Fe^2+^, Fe^3+^ and Cu^2+^ are typical examples of such “open shell” cations while Cu^+^ has a closed shell (all electrons are paired). These electronic structures are the source of the characteristic chemistry of the transition metal ions: they gain or lose electrons easily and so can participate in redox reactions (reduction/oxidation) inherent to many types of biological processes. If uncontrolled, however, such reactions are potentially toxic.

**Table 1:** Reactive oxygen and nitrogen species. These molecules can be classified conveniently as radicals, in which at least one unpaired electron is present, or as closed shell molecules in which all electrons are paired. The two forms can normally be easily distinguished experimentally: radicals are usually paramagnetic whereas closed shell molecules are always diamagnetic.

	Reactive Oxygen Species	Reactive Nitrogen Species
Radicals	Alkoxyl RO Hydroperoxyl HO_2_ Hydroxyl OH Peroxyl RO_2_ Superoxide O_2_^−^	Nitric oxide NO Nitrogen dioxide NO_2_
Closed shell molecules	Hydrogen peroxide H_2_O_2_ Hypochlorous acid HOCl Ozone O_3_ Singlet dioxygen ^1^O_2_ *Peroxynitrite OONO^−^	Dinitrogen trioxide N_2_O_3_ Nitronium ion NO_2_^+^ Nitrosyl cation NO^+^ Nitrous acid HNO_2_ Nitroxyl anion NO^−^ Nitryl chloride NO_2_Cl * Peroxynitrite OONO^−^

* This anion can be placed in either class.

The Zn^2+^ ion has a closed shell and rarely participates in redox reactions. However, it can bind typical biomolecules as a ligand and can thereby activate them for acid-base catalysis. The relative stabilities of Fe^3+^ and Fe^2+^ mean that the redox potential of the Fe^3+^/Fe^2+^ “couple” is able to catalyse many of the one-electron redox reactions needed in biology. Consequently, iron enzymes evolved for such duties in the reducing conditions of the primitive earth. The evolution of photosynthesis led to the highly oxidising conditions of the evolved earth and released copper from its sulfide ores. The more oxidising Cu^2+^/Cu^+^ couple is suitable for many redox processes under oxidising conditions and so copper redox enzymes have appeared in numerous important biochemical processes such as iron transport, erythropoiesis, melanin synthesis, mitochondrial respiration, glucose metabolism and antioxidant defences [19]. Examples include cytochrome c oxidase, ceruloplasmin, hephaesitin, dopamine β-hydroxylase, lysyl oxidase, tyrosinase and superoxide dismutase 1 (SOD1 [20]).

Uncontrolled copper is highly toxic due to its redox properties and its ability to displace other essential metals from their native sites [21]. Copper transporters, such as the P-type ATPases ATP7A and ATP7B [22], regulate the flux of copper across cell membranes and a series of soluble chaperones deliver the ion to the protein scaffold of enzymes. The Alzheimer’s disease-related amyloid precursor protein may participate in such processes [23].

Manganese has versatile redox chemistry and Mn^2+^, Mn^3+^ and Mn^4+^ are involved in a wide range of redox enzymes including the oxygen-evolving centre. In human systems, it acts as a cofactor in hydrolases, lyases, isomerases and ligases as well in transferases, lectins and integrins. Manganese is transported from the intestine and across the blood brain barrier by the transporter DMT-1. Mitochondrial superoxide dismutase 2 employs Mn^3+^/Mn^2+^ couple and this Mn-SOD2 is utilised by all aerobic organisms on Earth [24,25] except for a small number of bacterial species.

## 4. Superoxide Dismutase

Transition metal ions participate in a variety of antioxidant defence systems. For example, Cu^2+^ and Mn^2+^ are essential cofactors in the enzymic activity of the SOD enzymes. SOD1 also contains Zn^2+^ in a structural (non-catalytic) role and employs the catalytic Cu^2+^/Cu^+^ couple, *i.e*., copper cycles between its +2 and +1 oxidation states as it first reduces superoxide (O_2_^−^) to hydrogen peroxide (H_2_O_2_) and then oxidises it to dioxygen. It disproportionates or “dismutates” superoxide radicals in that the first radical is reduced while the second is oxidised.


Enzyme-Cu^2+^ + O_2_^−^ → Enzyme-Cu^1+^ + O_2_
Enzyme-Cu^1+^ + O_2_^−^ + 2 H^+^ → Enzyme-Cu^1+^ + H_2_O_2_
2 O_2_^−^ + 2 H^+^ → H_2_O_2_ + O_2_  Net SOD reaction

In this enzyme, the copper ion is bound to the protein by the sidechains of multiple histidine residues (ligands). These tune the Cu^2+^/Cu^+^ couple redox potential to that required to catalyse the net SOD reaction. This is the way in which different types of enzymes can “tune” the same metal ion for different reactions [26]. The Mn^3+^/Mn^2+^ couple is tuned by the protein ligands in SOD-2 to catalyse the net SOD reaction.

Enzyme-Mn^3+^ + O_2_^−^ → Enzyme-Mn^2+^ + O_2_
Enzyme-Mn^2+^ + O_2_^−^ + 2 H^+^ → Enzyme-Mn^3+^ + H_2_O_2_
2 O_2_^−^ + 2 H^+^ → H_2_O_2_ + O_2_  Net SOD reaction

In each of these examples, the product H_2_O_2_ is converted by catalase enzymes to water and O_2_. This again is a disproportionation reaction but involves two-electron transfers. Alternatively, H_2_O_2_ is converted to water by glutathione peroxidase in a two-electron reduction [27].

## 5. Fenton and Haber-Weiss Reactions

As many transition metal compounds contain unpaired electrons, they can be regarded themselves as free radicals. They are normally handled efficiently by membrane pumps and chaperones that ensure they are tightly bound for intracellular storage or as co-factors in enzymes. If improperly directed and left “free” (*i.e.*, not bound to sites of high affinity), transition metals are able to participate in such deleterious chemistry as Fenton and Haber-Weiss redox reactions that produce toxic hydroxyl radicals (OH**)**.

Fe^2+^ + H_2_O_2_ → Fe^3+^ + OH**^−^** + OH   Fenton reaction

The one-electron redox couples M*^n^*^+1^/M*^n^*^+^ (M = transition metal) in the general equation below typically represent Fe^3+^/Fe^2+^ and Cu^2+^/Cu^+^.

M*^n^*^+1^ + O_2_^−^ → M*^n^*^+^ + O_2_
M*^n^*^+^ + H_2_O_2_ → M*^n^*^+1^ + OH**^−^** + OH
O_2_^−^ + H_2_O_2_ → O_2_ + OH**^−^** + OH   Net Haber-Weiss reaction

These reactions produce OH radicals by single-electron interchange utilising the transient oxidation states of the available transition metal ion. The problem is that two electrons are needed to safely reduce H_2_O_2_ to 2 OH**^−^** (*i.e*., to water; as catalysed by glutathione peroxidase) but the metals can supply one electron only.

The highly oxidising OH radical is capable of generating further radicals in chain reactions leading to greater cellular damage [28]. While oxidatively modified proteins, lipids and nucleic acids are detected in the brain tissue from patients of most forms of neurodegenerative diseases, time course studies of mouse models of these diseases are yet to definitively determine if these modifications are a causative factor or simply an epiphenomenon of disease [29,30]. If the causative factor leads to impaired homeostasis of iron and copper, then free ions can induce the production of OH radicals or themselves displace other nutrient metals from their native sites.

## 6. Prion Protein Possesses Antioxidant Activity

While PrP^C^ has been shown capable of acting as a non-specific quencher of free radicals [31], published data from various groups support that PrP^C^ possesses SOD-like antioxidant capacity [32]. However, apparently conflicting data exists [33,34]. Homogenates derived from the brain tissue of prion protein knockout (PrP%) mice have been shown to possess less total SOD activity and greater Mn-SOD2 activity compared to wild-type controls [35]. Wong *et al*. [36] were able to show that homogenates of naïve, healthy brain tissue immuno-depleted of PrP^C^ similarly displayed reduced total SOD activity compared to non-depleted controls, while the specific activities of the Cu/Zn-SOD1 and Mn-SOD2 enzymes were unaltered.

Cultured neurons from the brains of PrP% mice incur greater cellular damage and death when exposed to superoxide anions [37] and hydrogen peroxide [38] as well as when elevated levels of manganese [39] and copper [40] were added to growth media in comparison to cognate wild-type control cultures. In accord with these findings, Wong *et al*. [41] demonstrated that preparations of prion protein purified from the brains of healthy mice (PrP^C^) exhibited roughly ten-fold the total SOD activity when compared with similar preparations derived from scrapie-infected brain tissue (PrP^C^ plus PrP^Sc^). It may be that the conversion to PrP^Sc^, or perturbations to cellular activity occurring during the process of infection, act to inhibit the normal SOD-like activity of PrP^C^ [42] although it is likely that the result of conformational changes to PrP^C^ molecules, and the generation of altered PrP^Sc^ isoforms, leads to the loss of intrinsic PrP^C^ antioxidant capacity. This diminished activity may relate in part to alterations in copper binding capacity during this conformational change [43]. Indeed, the SOD-like activity of recombinant PrP^C^ has been shown to rely on the presence of copper ions [44] and the SOD-like activity of total prion protein preparations isolated from the brains of an experimental mouse model of prion infection was demonstrated to decrease across the incubation period concomitant with a reduction in the amount of copper bound [45]. Further, there is evidence that suggests an inverse correlation between the expression levels and antioxidant activities of Mn-SOD2 and PrP^C^ under normal conditions. In addition, mitochondrial preparations from PrP% mice have been shown to exhibit elevated levels of Mn-SOD2 expression and activity [46]. Similar preparations from transgenic mice over-expressing PrP^C^ displayed diminished levels of Mn-SOD2 activity [47]. The evidence suggests that mitochondria may benefit from the antioxidant activity of PrP^C^.

## 7. Oxidative Stress Contributes to Prion Pathogenesis

Oxidative stress is apparent in the numerous animal models of prion infection representing various strains of prions introduced into a diverse range of experimental animals. This often includes diminished (Cu/Zn SOD or Mn-SOD2) activity or SOD-like activity [40,45] and elevated radical-mediated lipid peroxidation [13,16,17,41,48]. Lee *et al*. [48] also detected a significantly greater rate of superoxide radical generation in the brains of 87 V scrapie-infected mice when compared with uninfected control brain tissue. Similar changes have been detected at autopsy examining the brains of sporadic CJD patients where evidence of heightened lipid peroxidation has been shown [49]. The involvement of hydroxyl radicals in the generation of lipid peroxidation events is well recognised and the presence of this form of oxidative damage in the brain is consistent with transition metal ions catalysing Fenton and Haber-Weiss reactions [50]. Other forms of oxidative damage have been reported including modifications to proteins [14,41] and nucleic acids [15,51] as well as reports of increased nitrosative damage to both [12,14,41]. Overall, these reports indicate that increased reactive oxygen and nitrogen species are generated during the pathogenesis of prion disease. It appears that this oxidative/nitrative stress is increased with diminishing PrP^C^ antioxidant contribution during the onset of disease leading to heightened oxidative/nitrosative damage in the PrP^Sc^-diseased brain. In this way, free radical and reactive oxygen and nitrogen species damage could play a significant contributing factor in the pathogenesis of prion diseases.

## 8. Antioxidant Therapy Combating Models of Prion Disease

While investigations into the efficacy of quinacrine, shown to possess antioxidant capacity [52] and to demonstrate anti-prion activity *in vitro* [53,54], have unfortunately returned negative results in both animal model studies [55] and human trials [56], a more specific and potent antioxidant compound has provided foundation for the further development [57,58,59] of antioxidant therapies targeting prion (and other related) diseases. EUK-189, a Mn-SOD2 mimetic with reported catalase activity, was trialled in a study utilising a mouse-adapted Gerstmann-Sträussler-Scheinker syndrome (GSS) prion (termed M1000) model of infection. Mice administered EUK-189 survived significantly, albeit modestly longer than untreated controls and, through comparative histological analyses, the EUK-treated mice were shown to display a significantly reduced vacuolar lesion burden in specific regions of the brain [60].

## 9. Transition Metals Contribute to Neurodegeneration

While the action of many different transport and storage molecules contribute to the maintenance of neuronal transition metal homeostasis within the brain, unmitigated exposure or dysregulation of these potentially harmful elements may overwhelm these mechanisms and lead to perturbed distribution of certain metals. Exposure to heavy metals such Mn^2+^ [61], Hg^2+^, Cu^2+^, Zn^2+^, Pb^2+^, and Al^3+^ is recognised to cause neurodegeneration. The normal distribution of essential Mn, Cu, Zn and Fe is altered resulting in the accumulation of metals in specific regions of the brain. An example is manganism, a disorder displaying Parkinson’s disease-like symptoms, that is induced by toxic accumulation of Mn in the globus pallidus of the brain [62]. In addition, a number of transition metals have been implicated as playing a role in the pathogenesis of common neurodegenerative diseases contributing, for example to the aggregation process of amyloidogenic proteins in Alzheimer’s and Huntington’s diseases. While the particular proteins and transition metals involved differ for each disease, their association may trigger a conformational change to one more capable of aggregation or stabilise a misfolded isoform of the protein. This may result in concatenation and the ordered formation of amyloid fibrils [63]. Arguably, the prion protein is the most extensively studied transition metal-binding amyloidogenic protein implicated as a contributor to neurodegenerative disease [64,65]. Recent reports have demonstrated that PrP^C^ also mediates the toxic effects of Aβ oligomers in Alzheimer’s disease [66,67,68,69].

## 10. Transition Metal Participation in Prion Disease

**Figure 2:**
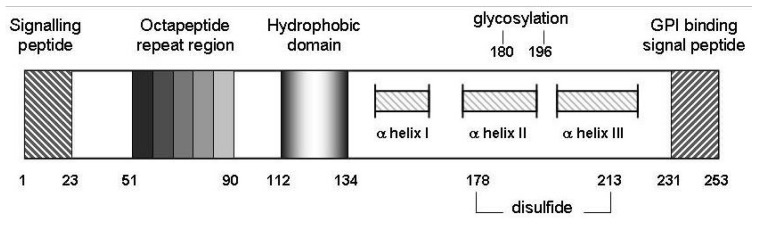
Schematic representation of murine prion protein (PrP^C^). Wild-type PrP^C^ contains 4 tandem repeats approximating an octapeptide sequence, an hydrophobic core from amino acids 112 to 134 as well as 2 potential sites for glycosylation at N residues 180 and 196. Regions from 145 to 155, 175 to 193 and 200 to 219 from three alpha-helical structures, and helices II and III are disulfide bridged between C residues 178 and 213 [65]. Not to scale.

The octameric repeat region of PrP^C^ (Figure 2) has been shown to bind 4 (and, in some conditions, up to 6 [70]) atoms of copper [35] while an additional binding site has been suggested to utilise histidine residues 95 and 110 (mouse sequence, 111 in the human sequence [71]. There have been many reports of the binding affinity of Cu^2+^ for the prion protein with estimates scattered from 10^−6^ to 10^−14^ M [72,73,74]. Within the octameric repeat region, it is the histidine in the repeated HGGGW sequence that is considered the primary coordinating residue involved in copper binding. [75,76] The region can bind multiple Cu^2+^ ions with stoichiometries and modes varying with Cu content and medium pH [77]. The additional site, incorporating His residues upstream of the PrP^C^ octarepeat region is reported to have a greater affinity for Cu^2+^ than does the octarepeat region [78,79]. The affinity of the octomeric repeat region for *Cu^+^* does not appear to have been assessed. This is needed to determine whether the binding in this region can act as catalysts: a binding site must stabilise *both* Cu^2+^ and Cu^+^ to be an effective catalyst. Otherwise, the metal ion dissociates during turnover.

Studies of the influence that Cu^2+^ can have on the conformation of the prion protein have provided somewhat conflicting results although differences in the buffers used for *in vitro* analyses make the interpretation and comparison of results difficult; there is little consensus among protocols for the preparation of copper-bound PrP in studies of the effect the metal ion has on PrP conformation. When present at relatively high concentrations, some buffers can compete with PrP for Cu^2+^ and lead to skewed results.

Cu^2+^ is reported to promote conversion of folded aged full-length recombinant mouse PrP to an isoform similar to PrP^Sc^ [80]. It has been shown to convert PrP^C^ purified from healthy mice brains to a protease-resistant isoform that, however, is structurally dissimilar to PrP^Sc^ [81]. Copper has been reported to inhibit conversion of full length recombinant PrP by stabilising a protease-resistant, non-amyloidogenic form of PrP. However, the same study found that when Cu^2+^ was added to pre-formed fibrils, protein aggregation and protease resistance was found to be significantly increased [82]. The apparent complexity of the influence of copper on the conversion of PrP^C^ into a more pathogenic isoform underscores the need to define the precise role of copper in both healthy and disease settings. It may be necessary for investigators to establish standard conditions for a particular strain of prion protein while following common protocols for its interaction with copper.

There is evidence of transition metal dyshomeostasis in the brains of mice infected with scrapie. The effect may be partly due to an altered conformation of the prion protein with diminished capacity to coordinate copper. Studies have also demonstrated significantly elevated levels of manganese in prion-infected brains when compared to non-infected controls [49,83]. A report examining purified prion protein harvested periodically across the time-course of disease [45] suggests that this is likely to be due to PrP^Sc^ binding manganese in preference to copper [43]. While conformational change to the diseased isomer appears to influence cellular uptake of Mn^2+^ [84], PrP^C^ is thought to act as a copper transporter; in neuronal culture experiments PrP^C^ was shown to promptly internalise copper [85,86]. PrP^C^ expression has also been linked to copper’s influence on depolarisation of the synapse [87].

Ablation of the prion protein in PrP% mice resulted in changes to a range of proteins (such as Atox1) involved in homeostatic regulation of copper levels [88]. Alterations in the capacity of neuronal copper transport can result in severe neurological disease. Increased copper accumulation can lead to Wilson’s disease and decreased copper to Menke’s disease [89,90]. Uncontrolled cellular copper concentrations can also cause mitochondrial dysfunction and lead to oxidative stress due to free copper reacting to generate free radicals via the Fenton and Haber-Weiss reactions, as described above. Uncontrolled copper can also interfere with the roles of other nutrient metals.

The prion protein has been shown both computationally and empirically to be capable of binding transition metal ions in addition to copper [91]. The most extensively studied appears to be manganese with many groups establishing a pathogenetic involvement in prion diseases. Isothermal titration calorimetry identified two Mn^2+^ binding sites in PrP with affinities similar to other known Mn-binding proteins [43]. The highest-affinity binding site incorporated His-95 (mouse PrP sequence) of the copper binding site outside the octarepeat region. Mn^2+^ could apparently replace Cu^2+^ at this site and induce an altered conformation typical of PrP^Sc^-associated structures [43].

Others have demonstrated manganese to be involved in the pathogenesis of prion disease including studies of tissue from a variety of species. Elevated manganese levels have been observed in the CNS and blood tissue from human patients suffering from prion disease [92]. There was an elevated level of Mn found associated with PrP purified from the brains of sporadic CJD patients when compared with that obtained from normal brains [49]. Higher zinc and lower copper levels were also found. Similar changes in PrP-metal association have been observed in scrapie- and bovine spongiform encephalopathy-afflicted livestock prior to the onset of the symptomatic stage of disease [93]. As such, the highly reproducible changes to the levels of blood Mn and Cu during disease onset have attracted interest as a possible early diagnostic tool especially within the livestock farming industry [93].

## 11. Prion Protein Aggregation in Prion Disease

Binding of Mn^2+^ induces the prion protein to adopt a structure displaying many of the biochemical properties of PrP^Sc^ [94,95,96]. Upon aging, this form was shown to be relatively protease resistant and to have lost antioxidant function compared to the Cu^2+^-isoform [94,97]. It is also more prone to participate in the assembly of ordered amyloid fibrils [98], with the latter congruent with observations that manganese addition can also promote higher order aggregation events [43,96]. Manganese association has been shown to promote the *in vitro* conversion of PrP in the *in vitro* protein misfolding cyclic amplification (PMCA) method of PrP^Sc^ propagation. This effect was inhibited by the addition of Cu^2+^ [95]. The observation that Mn-treatment of normal hamster brain homogenate was able to enhance the efficiency of PrP^C^ conversion in the presence of a catalytic amount of PrP^Sc^ seed helps support the notion that Mn^2+^ may facilitate the *de novo* generation of PrP^Sc^ *in vivo* [99,100].

**Figure 3:**
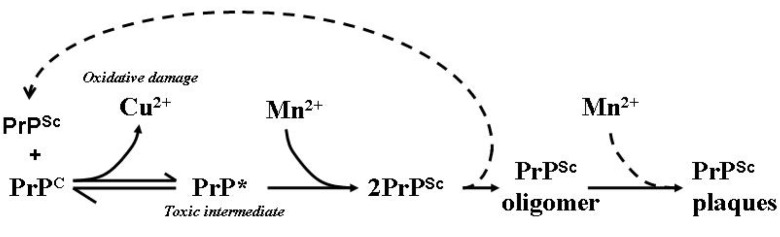
Model of the involvement of Mn in the generation of PrP^Sc^ isoforms, aggregates and plaques. PrP^Sc^ is able to influence the conformation of PrP^C^ in a template-driven manner. This altered conformation of PrP^C^ loses its affinity for Cu while increasing an affinity for Mn facilitating the stabilisation and accumulation of PrP^Sc^ and the eventual formation of PrP plaques as aggregated protein dumps. Free Cu is able to participate in deleterious redox reactions which can generate free radicals capable of damaging cellular macromolecules such as lipid membranes, proteins and DNA. PrP* represents the proposed toxic intermediate; dotted lines represent assumed associated reactions [83].

Manganese can alter the structure of PrP in different ways depending on the mode of association. Circular dichroism (CD) spectroscopy has detected differences in β-sheet content depending on whether manganese was present during refolding from a denatured form or if manganese was reacted with PrP already refolded in the presence of copper (Mn-attacked Cu-PrP). This experiment represents a more physiologically relevant paradigm for generation of PrP^Sc^ isoforms (Figure 3). The CD spectrum for Mn-attacked Cu-PrP was shown to contain less α-helical and greater β-sheet structure than Cu-refolded PrP in the absence of manganese, and this manganese-attacked conformer promoted the aggregation of non-metallated, apo-PrP in spectrophotometrically -monitored turbidity assays [42]. As well as manganese, other metals have been demonstrated to influence the aggregation of PrP. The PrP 106–126 peptide was shown to bind either copper or zinc leading to aggregation and cellular toxicity [101]. The toxic effect of this metallated peptide was shown to be reversible upon the addition of highly specific metal chelators [102].

## 12. Therapeutic Manipulation of CNS Metals in Prion Disease

As stated previously, numerous publications implicate oxidative stress [12,13,14,15,16,17] and transition metal imbalance [99,103,104] in the pathogenesis of prion disease. The brains of moribund prion-infected mice [105] and autopsied GSS patients [49] display significantly reduced Cu^2+^ as well as heightened Mn^2+^ concentrations [45,106]. These disturbances are accentuated in preparations of neuronal synaptosomes from the brains of scrapie-inoculated mice, with significant changes occurring as early as 100 days post-inoculation [104]. At this point, the first signs of typical histological changes are becoming apparent by routine examination. Utilisation of laser microdissection (PALM) would offer further insight into the role of these metals in pathogenesis as it allows the precise isolation of PrPSc-containing plaques (Figure 1B) from the adjacent tissue to determine the changes in metals’ concentrations over the time-course of disease evolution.

Studies have investigated selective chelators as therapeutics targeting accumulation of Cu^2+^ or Mn^2+^ in the brain. They have resulted in significant survival outcomes although attempts at combination therapy have not been reported to date. The relatively selective copper chelator D-penicillamine was trialed in a mouse model of prion disease achieving an approximately 10% extension to survival times when compared with the survival periods of control mice resulting from low-dose inoculation but there was no benefit observed among mice infected with a higher dose [105]. Similarly, a study of the relatively selective manganese chelator CDTA, in a mouse model of intracerebral M1000 prion infection, achieved an extension to survival of approximately 10% but also only in mice infected with low-dose inocula [83]. As both studies removed their targeted metals selectively but not specifically nor completely, it is difficult to generate firm conclusions regarding the direct pathogenic contributions of copper and manganese in prion disease. Significant concentrations of these metals were shown to remain in the brains of treated mice in both studies. Other transition metal ions have also been shown to enhance formation of PrP^Sc^-like conformations of PrP [99] and such effects could occur at lower levels of copper and manganese during these chelation experiments.

Clioquinol (CQ [107]), a Cu/Zn chelator of low affinity, has shown considerable benefit in preventing the formation of Aβ-containing plaques in a mouse model of Alzheimer’s disease [108]. It also provided positive results in trials of Parkinson’s disease [109] and has been tested as a metal-attenuating therapeutic in animal models of TSE. Ponti *et al*. [110] reported a 60% extension to the survival period of prion-infected mice treated orally with 7.5 mg/kg CQ per day when compared to untreated controls. CQ appears to be able to maintain normal brain function by assisting in the maintenance of transition metal—particularly zinc and copper—homeostasis [106]. However, while beneficial effects have been observed in mice inoculated intraperitoneally, benefit has yet to be reported for CQ-treated mice following intracerebral prion inoculation ([110] Brazier MW, unpublished results). The drug apparently offers benefit by slowing the rate of PrP^C^ to PrP^Sc^ conversion thereby extending incubation periods. This appears to also allow for aggravation of neuropathologic changes, as compared to untreated controls that succumbed to illness significantly earlier [110].

Investigations focusing on the manipulation of systemic copper and manganese concentrations through altered dietary intake during experimental prion disease incubation periods have provided some evidence that these metals have an influence on the course of disease, although there are some inconsistencies. Mice were found to display reduced PrP^C^ expression when maintained on a copper–depleted diet. Reducing available PrP^C^ substrate has been shown to be successful in slowing the propagation of PrP^Sc^ [111] but mice fed a diet specifically reduced in copper reached the terminal stage of disease significantly earlier than control mice fed a standard diet [104]. A copper-depleted diet during the scrapie-prion incubation period in another experiment led to mice developing significantly more vacuolar lesions and displaying heightened astroglial activation in particular regions of the brain [112]. These effects are likely due to heightened oxidative stress resulting from a reduction of SOD activity [2,60,104]. In reciprocal experiments, a diet high in copper delayed the onset of scrapie symptoms in infected mice [113] and extended the incubation period in comparison to normal-diet controls [104]. The brains of mice fed a manganese-rich diet were shown to express PrP^C^ to a greater extent with mice fed a similar diet during the disease incubation period shown to have increased neuronal loss and significantly more PrP-containing plaques when compared to control mice fed normal levels of manganese [112]. However, a manganese-rich diet has also been reported to have no significant influence on the incubation period of experimental prion disease [104].

## 13. Conclusions

Oxidative damage and transition metal dyshomeostasis in the brain appear to be inevitable consequences of prion disease although their precise and relative pathogenic contributions remain to be fully resolved. Nonetheless, sufficient consistency exists in reported results published by various laboratories investigating prion disease to underscore the likelihood that transition metal dyshomeostasis does indeed play a role in pathogenesis, probably through promoting the misfolding of PrP^C^ to abnormal conformers and/or promoting concatenation of the latter. The proposed conformational alteration of PrP^C^, that may occur as Cu^2+^, is released during disease evolution (Figure 3) is consistent with the notion that brain transition metal dyshomeostasis may also directly or indirectly contribute to pathogenesis through promoting free radical generation and heightened oxidative damage. The relatively minor benefit attained from antioxidant therapy suggests that O_2_^.−^ radicals may not be the major cause of the frequently-reported oxidative damage observed in the brains of patients and experimental animals infected with prion disease or that it is only a small population of cells that are affected in this manner. Examination of laser microdissection and pressure catapult (LMPC or PALM)—isolated cells from specific regions of damage will help considerably in the quest to understand *in vivo* cell damage. Data also implicates manganese as a facilitator in the conformational change of monomeric PrP^C^ to the aggregation-prone, disease-associated isomer PrP^Sc^, as well as in downstream events which promote the aggregation of pre-formed multimers into higher order aggregates. The benefits resulting from the specific reduction of brain Mn by chelation therapy may have been limited by the ability of other metal ions to associate with the prion protein and continue to generate pathogenic isoforms similar to PrP^Sc^ capable of sustaining pathogenesis.
